# Effect of Imperatorin on the Spontaneous Motor Activity of Rat Isolated Jejunum Strips

**DOI:** 10.1155/2015/614849

**Published:** 2015-07-08

**Authors:** Marta Mendel, Krystyna Skalicka-Woźniak, Magdalena Chłopecka, Natalia Dziekan

**Affiliations:** ^1^Division of Pharmacology and Toxicology, Faculty of Veterinary Medicine, Warsaw University of Life Sciences, 8 Ciszewskiego Street, 02-786 Warsaw, Poland; ^2^Department of Pharmacognosy with Medicinal Plants Unit, Medical University of Lublin, 1 Chodzki, 20-819 Lublin, Poland

## Abstract

Imperatorin, a psoralen-type furanocoumarin, is a potent myorelaxant agent acting as a calcium antagonist on vascular smooth muscle. Its effects on other types of smooth muscle remain unknown. Therefore, the aim of this study was to investigate the hypothesized myorelaxant effect of imperatorin on gut motor activity and, possibly, to define the underlying mechanism of action. Imperatorin was made available for pharmacological studies from the fruits of the widely available *Angelica officinalis* through the application of high-performance countercurrent chromatography (HPCCC). Imperatorin generated reversible relaxation of jejunum strips dose-dependently (1–100 *μ*M). At 25 and 50 *μ*M, imperatorin caused relaxation comparable to the strength of the reaction induced by isoproterenol (Isop) at 0.1 *μ*M. The observed response resulted neither from the activation of soluble guanylate cyclase, nor from *β*-adrenoreceptor involvement, nor from Ca^2+^-activated potassium channels. Imperatorin relaxed intestine strips precontracted with high potassium concentration, attenuated the force and duration of K^+^-induced contractions, and modulated the response of jejunum strips to acetylcholine. The results suggest that imperatorin probably interacts with various Ca^2+^ influx pathways in intestine smooth muscle. The types of some calcium channels involved in the activity of imperatorin will be examined in a subsequent study.

## 1. Introduction

**Figure 1:**
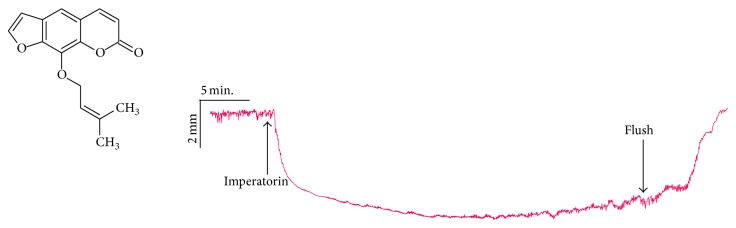
Structure of imperatorin and sample recording of rat isolated jejunum strip's reversible myorelaxation caused by imperatorin (75 *μ*M).

Imperatorin (Figure 1) is a naturally occurring furanocoumarin which is widely distributed in medicinal plants from the Apiaceae (formerly Umbelliferae) family. Due to its widespread occurrence, imperatorin was employed in several pharmacological studies aimed at understanding its effects on human and animal health. Imperatorin exerted an anxiolytic effect, improved different stages of memory processes [1], and protected against memory impairments induced by scopolamine, possessing antioxidant properties [2]. Imperatorin acts also as an anti-inflammatory [3], antiproliferative [4], anticonvulsant [5], acetyl- and butyrylcholinesterase inhibitor, antioxidant [6], antibacterial and anticoagulant [7], and vasodilatatory [8, 9] agent.

According to studies conducted on vascular smooth muscle [8, 9], imperatorin inhibits Ca^2+^ influx through voltage-sensitive calcium channels, and thus it can be described as a calcium antagonist. Compounds belonging to this group of pharmacological agents affect various body functions, including muscle contractility, gland secretion, neurotransmitter release, gene expression, and platelet cell activity. Indeed, imperatorin acts as a calcium channel blocker in rat pituitary cells [10], mouse myocardial cells [11], and various arteries, for example, rabbit thoracic aorta [12]. Since it was established that imperatorin influences the contractility of vascular smooth muscle, it was interesting to explore its effect on gut smooth muscle. Thus, the aim of the present study was to determine the response of isolated rat intestinal strips to imperatorin treatment and to investigate the underlying mechanism of action. Since rat jejunum preparations were previously used to evaluate the calcium blocking activity of different compounds [13], the study also included verification of the possible effects of imperatorin as a calcium blocker.

Like many natural products, imperatorin is not routinely available in large quantities for pharmacological studies. Therefore, based on previous studies [14, 15], high-performance countercurrent chromatography (HPCCC) was applied as an efficient tool for the isolation of imperatorin from the fruits of the widely occurring plant,* Angelica officinalis* Hoffm. (Apiaceae), which is used in traditional medicine for digestive problems, including gastric ulcers and stomach cramps, and has been shown to stimulate gastric and pancreatic secretions and to possess hepatoprotective activity [16, 17].

## 2. Materials and Methods

### 2.1. Chemicals

Acetylcholine chloride, dimethyl sulfoxide (DMSO), isoproterenol hemisulfate (Isop), methylene blue, propranolol, tetraethylammonium (Sigma Chemical Co., St. Louis, MO, USA), CaCl_2_ (Merck, Darmstadt, Germany), NaH_2_PO_4_ (Fluka Chemie, AG, Buchs, Switzerland), and all other salts needed for the preparation of the incubation media, that is, NaCl, KCl, MgSO_4_, NaHCO_3_, and glucose (POCh, Gliwice, Poland), were used for preparing experiments on isolated jejunum strips. Imperatorin was dissolved in 0.5% DMSO; all other substances were dissolved in modified Krebs-Henseleit solution. All solvents used for countercurrent chromatography were of analytical grade and purchased from Polish Reagents (POCh Gliwice, Poland). Methanol used for high-performance liquid chromatography (HPLC) was HPLC grade and was purchased from Merck (Darmstadt, Germany), and the water used was purified using a Millipore Laboratory Ultrapure water system (Simplicity system, Millipore, Molsheim, France).

### 2.2. Plant Material

Fruits of* A. officinalis* were collected in August, 2011, in the Medicinal Plant Garden of the Medical University of Lublin, Lublin, Poland. A voucher specimen (no. B2/7-9), representative of the collection, is deposited in the Department of Pharmacognosy, Medical University of Lublin, Poland. A sample (30 g) of dried and milled fruits was extracted three times (30 min. each time) under reflux with methanol to afford, after evaporation, an extract (3.71 g).

### 2.3. Extraction and Isolation

The high-performance countercurrent chromatograph used in this study was a Spectrum HPCCC, available commercially from Dynamic Extractions (Slough, UK), and a semipreparative coil with a capacity of 137 mL was used. The effluent from the coil was monitored by a UV detector at 254 nm (ECOM, Czech Republic). The two-phase solvent system used was composed of heptane : ethyl acetate : methanol : water in the ratio 6 : 5 : 6 : 5 (v/v) on the basis of the calculated partition coefficients values (*K*_*D*_). The upper phase (the stationary phase) was pumped into the semipreparative column. After the column was totally filled, the apparatus was rotated at 1600 rpm. At the same time, the lower mobile phase was pumped into the column at a flow rate of 6.0 mL/min. When equilibrium was reached and 30 mL of stationary phase was displaced, the extract (500 mg) dissolved in the same two-phase solvent system (6 mL) was injected. One-minute fractions were collected manually, and the separation was stopped after 45 minutes. In a single run, 35 mg of imperatorin was isolated. The identity and purity of imperatorin were confirmed by HPLC-DAD-ESI-TOF-MS analyses, as described previously [15]. The isolate was identified based on the mass spectra of reference compounds and MS data from the literature [18].

### 2.4. Isolation and Preparation of Rat Isolated Jejunum Strips

The experiments were conducted on intestine segments isolated from Wistar rats. The study was approved by the local ethics committee (approval number 8/2011). The animals were euthanized in chambers filled with carbon dioxide (CO_2_) [19]. Immediately after euthanasia, jejunum segments were incised and prepared as described previously [20]. All preparations were suspended in incubation chambers (5 mL) (Schuler Organ Bath, Hugo Sachs Elektronik-Harvard Apparatus, March-Hugstetten, Germany) filled with warmed (37°C), modified Krebs-Henseleit solution M K-HS (NaCl, 123.76 mM; KCl, 5 mM; CaCl_2_, 2.5 mM; MgSO_4_, 1.156 mM; NaHCO_3_, 14.5 mM; KH_2_PO_4_, 2.75 mM; and glucose, 12.5 mM) and bubbled with carbogen.

### 2.5. Registration of Motor Activity of Rat Isolated Intestine Strips

All experiments were carried out under isotonic conditions, under a load of 0.5 grams. The registration of the data was performed through PowerLab (ADInstruments, Sydney, Australia) and bridge amplifier (DBA, type 660, Hugo Sachs Elektronik-Harvard Apparatus, March-Hugstetten, Germany). Subsequently, the records were analyzed by Chart v7.0 program and Excel (MS Office XP Professional).

### 2.6. Experimental Design

The experiments were initiated with a 60-minute preincubation period. Subsequently, two reference substances in optimal doses (acetylcholine (ACh) of 1 *μ*M and isoproterenol (Isop) of 0.1 *μ*M) [21] were administered in order to verify the reactivity of the strips and were considered as the positive controls. The preparations were treated with DMSO at a concentration of 0.5%, and the reaction of the preparations to DMSO (0.5%) was considered a negative control response. Once the spontaneous activity stabilized, imperatorin was applied in a noncumulative manner in a concentration range of 0.001–100 *μ*M. At the end of each experiment, all jejunum strips were treated with ACh at the optimal concentration.

In order to verify the mechanism of action of imperatorin, the jejunum strips were preincubated for at least 5 minutes with methylene blue (100 *μ*M), tetraethylammonium (500 *μ*M), propranolol (10 *μ*M), or K^+^-rich M K-HS (containing 128.8 mM of KCl) and then exposed to imperatorin (50 *μ*M), or the application of imperatorin (50 *μ*M) was followed by treatment with Isop (0.1 *μ*M). In addition, in some experiments, imperatorin (50 *μ*M) was administered simultaneously with acetylcholine (1 *μ*M) or K^+^-rich M K-HS (HS containing 128.8 mM of KCl). Whenever K^+^-rich M K-HS was used, NaCl was replaced by KCl. All experiments were repeated at least 6 times.

### 2.7. Expression of the Results and Statistical Analysis

The results are expressed as a percent of the reaction caused by isoproterenol (0.1 *μ*M) or acetylcholine (1 *μ*M) in the case of the relaxant and contractile reactions, respectively. All data were analyzed using Statistica PL for Windows (v10.0). The results are expressed as mean values (±SD) of the average. Values of *p* ≤ 0.05 are considered to be significant. The following tests were employed in the statistical analysis: a one-way analysis of variance (ANOVA), Student's *t*-test, and the LSD Fisher test. The reaction of a jejunum smooth muscle strip to a tested compound was considered significant if its strength differed statistically from the force of the reaction to DMSO in a concentration of 0.5% (negative control).

## 3. Results

### 3.1. The Effect of Imperatorin on the Spontaneous Motor Activity of Rat Isolated Jejunum Strips

**Figure 2:**
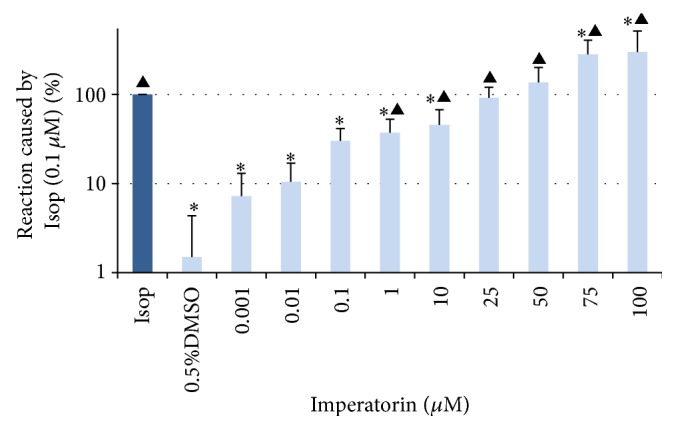
Myorelaxant effect of imperatorin dissolved in 0.5% DMSO on the spontaneous motoric activity of isolated jejunum strips. The results are measured as the maximal effect induced by imperatorin and expressed as % of the relaxation caused by isoproterenol applied in the reference dose 0.1 *μ*M. The relaxation provoked by isoproterenol in the reference dose is expressed as 100%. The results are expressed as mean from 6-7 independent experiments (±SD). ^**∗**^ *p* ≤ 0.05 versus Isop; ^▲^ *p* ≤ 0.05 versus DMSO, 0.5%.

Imperatorin, dissolved in 0.5% DMSO, caused significant alterations (maximal reaction, basal tone, AUC) in the spontaneous motor activity of rat isolated jejunum strips (Figures 1 and 2, Table 1). All intestine preparations incubated with imperatorin were affected in the same way, and the observed reaction was always myorelaxant in character. The strength of the reaction was dose-dependent in the concentration range 1 to 100 *μ*M (Figure 2). The application of imperatorin at 25 and 50 *μ*M caused relaxation comparable to the strength of the reaction induced by Isop (0.1 *μ*M) (91.7 ± 28.9 and 136.3 ± 64.5% of the reaction to Isop, resp.) (Figure 2). The two highest concentrations of imperatorin (75 and 100 *μ*M) clearly exceeded the magnitude of the myorelaxant response of isoproterenol. Imperatorin at 100 *μ*M resulted in a relaxation at a magnitude of 300.1 ± 216.5% of the reaction caused by Isop (Figure 2). It is noteworthy that even if the strips were incubated with imperatorin applied at the highest concentrations, the reaction was reversible. Flushing of the preparation with fresh M K-HS always resulted in a rapid return (within three minutes) to the basal spontaneous motor activity. The force of the reaction to the reference contractile agent (ACh) administered at the beginning and at the end of each experiment was comparable.

### 3.2. Myorelaxant Effect of Imperatorin in the Presence of Different Compounds

**Figure 3:**
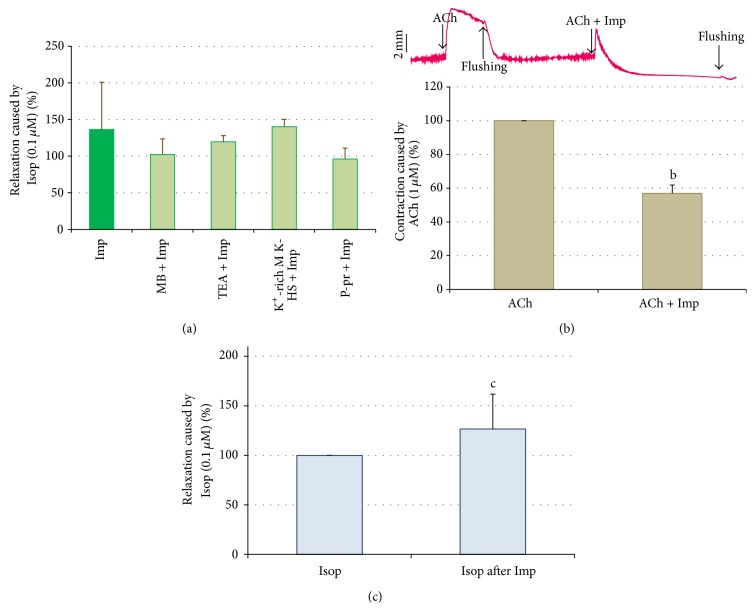
Effect of various agents on the myorelaxation of isolated jejunum strips caused by imperatorin (Imp, 50 *μ*M). (a) The effect of methylene blue (MB, 100 *μ*M), tetraethylammonium (TEA, 500 *μ*M), K^+^-rich M K-HS (128.8 mM of KCl), and propranolol (P-pr, 10 *μ*M) pretreatment on the reaction induced by imperatorin (50 *μ*M). (b) The effect of imperatorin (Imp, 50 *μ*M) on the contraction caused by acetylcholine (1 *μ*M). ACh: acetylcholine (1 *μ*M) application after 1-hour preincubation, ACh + Imp: simultaneous application of ACh (1 *μ*M) and Imp (50 *μ*M). (c) The effect of imperatorin (Imp, 50 *μ*M) on the myorelaxation caused by isoproterenol (0.1 *μ*M). Isop: the application of isoproterenol (0.1 *μ*M) at the beginning of each experiment, Isop after Imp: the application of isoproterenol (0.1 *μ*M) preceded by the administration of imperatorin (50 *μ*M). The results are expressed as % of the myorelaxation or contraction caused by isoproterenol or acetylcholine applied in the reference doses 0.1 *μ*M and 1 *μ*M, respectively. The reactions provoked by isoproterenol and acetylcholine in the reference doses are expressed as 100%. The results are expressed as mean from 6-7 independent experiments (±SD); ^a^ *p* ≤ 0.05 versus Imp; ^b^ *p* ≤ 0.05 versus ACh; ^c^ *p* ≤ 0.05 versus Isop. (The letter “a” refers to statistical significance versus imperatorin (50 *μ*M).)

The application of methylene blue (100 *μ*M), tetraethylammonium (500 *μ*M), or propranolol (10 *μ*M) did not generate any change of the spontaneous contractility of jejunum specimens. Pretreatment of jejunum smooth muscle strips with either methylene blue (100 *μ*M), tetraethylammonium (500 *μ*M), or propranolol (10 *μ*M) did not affect the reaction caused by imperatorin (50 *μ*M) (102.29 ± 21.32%, 119.60 ± 8.44%, and 96.05 ± 14.93%, resp., of the response to Isop). On the other hand, the myorelaxation which resulted from only imperatorin administration, and not preceded by any agent, amounted to 112.92 ± 40.42% of the response to Isop (Figure 3(a)).

**Figure 4:**
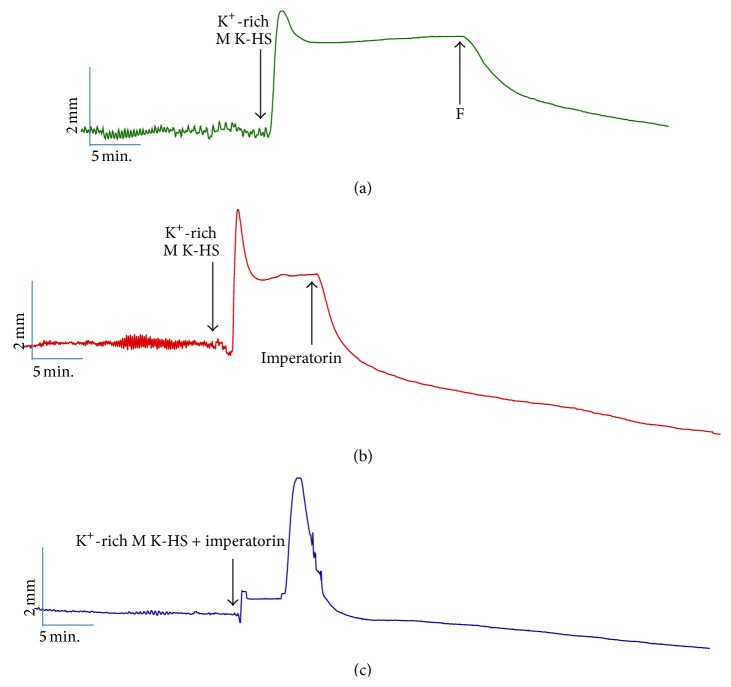
Sample recording of the isolated jejunum strips: (a) reaction to medium exchange to K^+^-rich M K-HS; (b) the response to the application of imperatorin (50 *μ*M) of K^+^-precontracted specimens; (c) the effect of simultaneous application of imperatorin (50 *μ*M) and K^+^-rich M K-HS on jejunum smooth muscle.

Imperatorin (50 *μ*M) caused myorelaxation of jejunum smooth muscle strips which were precontracted by incubation in K^+^-rich M K-HS. The relaxant response amounted to 140.17 ± 10.26% of the reaction induced by Isop (Figures 3(a) and 4(b)). The application of imperatorin (50 *μ*M) reduced the force and duration of the contractile response of the jejunum preparation evoked by K^+^-rich incubation medium (Figure 4(c)).

The simultaneous administration of imperatorin (50 *μ*M) and acetylcholine (1 *μ*M) prevented the full development of the contraction normally evoked by ACh and amounted to 56.97 ± 4.90% of the usual response to ACh (Figure 3(b)). In addition, the duration of the registered contraction of jejunum strips was reduced from 3 minutes to less than 1 minute.

The intestine preparation relaxed with imperatorin (50 *μ*M) was unchanged by the application of isoproterenol (0.1 *μ*M). The force of the Isop-induced relaxant reaction of jejunum smooth muscle previously treated with imperatorin amounted to 126.59 ± 35.19% of the response to Isop (Figure 3(c)).

## 4. Discussion

The results indicate that imperatorin strongly and reversibly affects the spontaneous motor activity of isolated rat intestine strips. The reaction induced by imperatorin always had a myorelaxant character and was dose-dependent. The reversibility of the reaction suggests that imperatorin does not damage jejunal smooth muscle and probably does not bind covalently with smooth muscle cells. Similarly, the unchanged strength of the ACh-induced contraction observed at the end of the experiments confirms the suggestion that the strong myorelaxation evoked by imperatorin is not a consequence of cell disruption.

The results presented herein are analogous to data regarding the effect of imperatorin on vascular smooth muscle. Imperatorin (1–100 *μ*M) caused a concentration-dependent relaxation of phenylephrine-precontracted arterial rings [9]. It also evoked a relaxation of rabbit corpus cavernosum when used in doses of 30 and 100 *μ*M [22]. According to Chiou et al., imperatorin is a more potent vasodilator than papaverine, a reference myorelaxant agent. Similarly, imperatorin used at the highest concentrations (75 and 100 *μ*M) causes stronger myorelaxation than isoproterenol, a reference relaxant agent employed in the presented study (Figure 2, Table 1). In addition, the myorelaxant effects on gastrointestinal smooth muscle of other furanocoumarins, for example, xanthotoxin and visnadine, and plant extracts rich in those compounds are well documented [23, 24]. A previous study from this laboratory showed that bergapten (5-methoxycoumarin) caused myorelaxation of intestine preparations in the concentration range of 0.0001–1 *μ*M. At higher doses, bergapten caused either relaxation or contraction of the smooth muscle [14].

In order to investigate whether the relaxation induced by imperatorin might be due to an interaction with nitric oxide pathways, the tissues were pretreated with methylene blue. Methylene blue inhibits soluble guanylate cyclase in smooth muscle, and, thus, it decreases tissue guanosine 3′,5′-cyclic monophosphate, which is probably the second messenger involved in NANC (nonadrenergic, noncholinergic) responses in gastrointestinal smooth muscle [25]. No change was observed in the force of imperatorin-induced relaxation in strips pretreated with methylene blue in comparison to untreated preparations (Figure 3(a)). This suggests that the reaction provoked by imperatorin does not result from the activation of soluble guanylate cyclase.

Propranolol was used to evaluate the contribution of the *β*-adrenoreceptor in imperatorin-induced relaxation of isolated jejunum strips. The obtained results (Figure 3(a)) indicate that the *β*-adrenoreceptor is not involved in the reaction evoked by imperatorin. This finding confirms the conclusions of He et al. [8] who claimed that propranolol did not attenuate the vasodilatation effect caused by imperatorin. To test the hypothesis that potassium channels may play a crucial role in the relaxant effects of imperatorin, tetraethylammonium chloride (TEA), a calcium-activated potassium channel inhibitor, was applied to the incubation chambers prior to the administration of imperatorin. TEA is considered to block the open potassium channels in jejunum smooth muscle [26]. Since this classical K^+^-channel inhibitor does not affect the reaction induced by imperatorin (Figure 3(a)), it is suggested that imperatorin does not affect Ca^2+^-activated potassium channels. Interestingly, tetraethylammonium slightly attenuated the relaxant response induced by imperatorin in rat mesenteric arteries [8]. Possibly, the discrepancy between these studies arises from the origin of the smooth muscle and the different functionality of the calcium-activated, potassium channels in vascular and gut smooth muscle.

Imperatorin relaxed intestine strips which were precontracted with a high potassium concentration (128.8 mM of KCl) and attenuated the force and duration of the contraction evoked by a K^+^-rich incubation medium (Figures 4(b) and 4(c), resp.) when the preparations were simultaneously treated with imperatorin and a high dose of potassium. This result is in accordance with findings referring to the actions on vascular smooth muscle [8, 9]. A high concentration of potassium in the incubation medium causes sustained contraction of jejunal smooth muscle (Figure 4(a)) due to membrane depolarization, followed by calcium influx through voltage-sensitive calcium channels (mainly of the L-type).

The observation based on the performed study allows for a comparison of the effect of imperatorin on intestinal smooth muscle with the effect caused by verapamil, a classical blocker of voltage-dependent calcium channels. Such mechanism of myorelaxant activity of imperatorin was also suggested in regard to vascular smooth muscle [8]. He et al. demonstrated that imperatorin inhibits the contraction induced by CaCl_2_ in Ca^2+^-free medium. Another possibility of increasing calcium influx in smooth muscle involves the activation of receptor-operated, calcium channels (ROCCs) stimulated by agonists acting on a range of G-protein-coupled receptors [27]. An example of ROCCs in rat jejunum smooth muscle is muscarinic receptors, predominantly subtypes M_2_ and M_3_ [28]. Acetylcholine, a nonselective agonist of muscarinic receptors (mAChRs), induces gastrointestinal smooth muscle contraction by activating calcium influx from extracellular space (M_2_ and M_3_ receptors) and by activating phospholipase C to liberate IP_3_ and subsequently to release calcium from the sarcoplasmic reticulum. The obtained results indicate that imperatorin does not prevent ACh-induced contraction of jejunum smooth muscle, which indicates that it does not act as a muscarinic antagonist. However, it does modulate the response of jejunum strips to acetylcholine. As presented in Figure 3(b), imperatorin diminished the magnitude of the reaction normally evoked by ACh and reduced the duration of the registered contraction (from 3 minutes to less than 1 minute). This observation possibly suggests that imperatorin affects the pathways related to mAChRs activation. An estimation of the possible interference between imperatorin and ROCCs in jejunum smooth muscle requires further studies. However, this finding is convergent with one of the postulated mechanisms of vasodilatation induced by imperatorin [8].

Eventually, the increase in intracellular calcium concentration might result from the activation of store-operated calcium channels (SOCCs) or release from intracellular stores [29]. The participation of SOCCs in imperatorin-induced vasodilatation was excluded by Zhang et al. [9]. In order to analyze the participation of SOCCs in imperatorin-induced myorelaxation of intestine, it would be preferable to apply a model of isolated jejunum smooth muscle cells and determine the effect of imperatorin on actively or passively depleted Ca^2+^ stores. However, it was out of the scope of the performed study and requires further explanation in the future. On the other hand, He et al. [8] observed that imperatorin significantly changes caffeine-evoked contraction in vascular smooth muscle. The effect of caffeine on intestine smooth muscle might be explained by multiple mechanisms that result in both transient contraction and inhibition of induced contraction [30, 31]. The heterogeneous effect of caffeine on gut smooth muscle decided to exclude the experiments aimed at studying the impact of imperatorin on Ca^2+^ release from intracellular stores from this study. Nevertheless, the effect of imperatorin on intracellular calcium release requires further investigation on intestinal smooth muscle preparations or cells.

## 5. Conclusions

The obtained results revealed that imperatorin significantly affects the spontaneous motor activity of rat jejunum strips. It causes very strong, reversible relaxation of the intestine smooth muscle. The observed effect of imperatorin probably results from the interaction with various Ca^2+^ influx pathways in intestine smooth muscle.

## Figures and Tables

**Table 1:** The effect of DMSO (0.5%), imperatorin (75 *μ*M), and verapamil (10 *μ*M) on the spontaneous motoric activity of rat isolated jejunum specimens.

	Basal tone	AUC
DMSO (0.5%)	−0.45 ± 4.92^*∗*#^	0.21 ± 4.16^*∗*#^
Imperatorin (75 *μ*M)	188.04 ± 50.54^*∗*▲#^	181.43 ± 31.65^*∗*▲^
Imperatorin (100 *μ*M)	208.41 ± 29.37^*∗*▲#^	237.03 ± 45.41^*∗*▲^
Verapamil (10 *μ*M)	179.62 ± 24.37^*∗*▲^	211.10 ± 41.17^*∗*▲^

The results are expressed as % of the relaxation caused by isoproterenol applied in the reference dose 0.1 *μ*M. The relaxation provoked by isoproterenol in the reference dose is expressed as 100%. The results are expressed as mean from 6-7 independent experiments (±SD). ^*∗*^ *p* ≤ 0.05 versus Isop, ^▲^ *p* ≤ 0.05 versus DMSO, 0.5%, and ^#^ *p* ≤ 0.05 versus verapamil.
